# Draft Genome Sequence of *Lactobacillus collinoides* CUPV237, an Exopolysaccharide and Riboflavin Producer Isolated from Cider

**DOI:** 10.1128/genomeA.00506-16

**Published:** 2016-06-09

**Authors:** Ana Isabel Puertas, Vittorio Capozzi, María Goretti Llamas, Paloma López, Antonella Lamontanara, Luigi Orrù, Pasquale Russo, Giuseppe Spano, María Teresa Dueñas

**Affiliations:** a Department of Applied Chemistry, University of Basque Country (UPV/EHU), San Sebastián, Spain; b Department of Agriculture, Food and Environment Sciences, University of Foggia, Foggia, Italy; c Department of Molecular Microbiology and Infection Biology, Centro de Investigaciones Biológicas (C.S.I.C), Madrid, Spain; d Council for Agricultural Research and Economics (CREA)-Genomics Research Centre, Fiorenzuola d'Arda (PC), Italy

## Abstract

*Lactobacillus collinoides* CUPV237 is a strain isolated from a Basque cider. *Lactobacillus collinoides* is one of the most frequent species found in cider from Spain, France, or England. A notable feature of the *L. collinoides* CUPV237 strain is its ability to produce exopolysaccharides.

## GENOME ANNOUNCEMENT

*Lactobacillus collinoides* CUPV237 is a strain isolated from a Basque cider at the Department of Applied Chemistry, Faculty of Chemistry (University of the Basque Country UPV/EHU, San Sebastián, Spain). In the Basque Country (Northern Spain), natural cider is produced in small factories using traditional techniques, normally using juices obtained from many varieties of fresh cider apples (bitter, bittersweet, and sweet) to obtain a balanced content of sugars, acids, and polyphenols, without the addition of extra sugar or CO_2_. Alcoholic and malolactic fermentations occur spontaneously due to the indigenous yeasts and lactic acid bacteria present in the musts (1–3). The microbial decarboxylation of l-malic acid into l-lactic acid is of great importance from an organoleptic point of view, and most of the lactic acid bacteria isolated from cider have this capacity (1, 4–7). *Lactobacillus collinoides* is one of the most frequent species found in cider from Spain, France, or England (5, 8–10). A notable feature of the *L. collinoides* CUPV237 strain is its ability to produce exopolysaccharides.

Using the TruSeq DNA sample prep kit FC-121-1001 according to the manufacturer’s instructions, 2 µg of genomic DNA of *L. collinoides* was subjected to library preparation. Whole-genome sequencing was performed using the Illumina GAIIx platform at the CREA Genomics Research Centre (Fiorenzuola d’Arda). A total of 8,107,203 paired-end reads ranging from 90 to 115 bp in length were *de novo* assembled using CLC Genomics Workbench v7.0. The assembly resulted in 127 contigs with an *N*_50_ length of 70,285 bp. The shortest contig was 203 bp and the longest was 243,224 bp. The draft genome consists of 3,707,616 bp. The genome sequence was annotated using the NCBI Prokaryotic Genome Annotation Pipeline (http://www.ncbi.nlm.nih.gov/genome/annotation_prok).

### Nucleotide sequence accession numbers.

This whole-genome shotgun project has been deposited at DDBJ/ENA/GenBank under the accession number JYDC00000000. The version described in this paper is version JYDC01000000.
